# An fMRI Study of Local Synchronization in Different Subfrequency Bands during the Continuous Feedback of Finger Force

**DOI:** 10.1155/2015/273126

**Published:** 2015-06-09

**Authors:** Hang Zhang, Zhong-Zhan Gao, Yu-Feng Zang

**Affiliations:** ^1^Paul C. Lauterbur Research Center for Biomedical Imaging, Shenzhen Institutes of Advanced Technology, Chinese Academy of Sciences, Shenzhen 518055, China; ^2^Center for Cognition and Brain Disorders and the Affiliated Hospital, Hangzhou Normal University, Hangzhou 310015, China; ^3^Zhejiang Key Laboratory for Research in Assessment of Cognitive Impairments, Hangzhou 310015, China

## Abstract

Conventional functional magnetic resonance imaging (fMRI) studies on motor feedback employ periodical blocked paradigm which does not allow frequency analysis of brain activity. Here, we carried out an fMRI study by using a continuous paradigm, that is, continuous (8 min) feedback of finger force. Borrowing an analytic method widely used in resting-state fMRI studies, that is, regional homogeneity (ReHo), we compared the local synchronization in some subfrequency bands between real and sham feedback, and the subbands were defined as Slow-6 (0.0–0.01 Hz), Slow-5 (0.01–0.027 Hz), Slow-4 (0.027–0.073 Hz), Slow-3 (0.073–0.198 Hz), and Slow-2 (0.198–0.25 Hz). Our results revealed that the five subfrequency bands of brain activity contributed to the changes of ReHo between real and sham feedback differently, and, more importantly, the changes in basal ganglia were only manifested in Slow-6, implicating the fact that ReHo in ultraslow band may be associated with the functional significance of BG, that is, motor control. These findings provide novel insights into the neural substrate underlying motor feedback, and properties of the ultraslow band of local synchronization deserve more attention in future explorations.

## 1. Introduction

The motor feedback is a technique that enables participants to effectively regulate some kinetic parameters such as muscle force [1], speed [2], and gestures [3]. It exhibits benefits in improving some motor functions like the stand balance [4], finger force [5], and bimanual coordination [6] and also subserves the motor function rehabilitation for the patients with brain disorders of Parkinson disease [7], brain damage [8], chronic stroke [9], and so forth. These clinical values prompt more and more investigations on the neural substrates underlying the motor feedback.

Neuroimaging investigations intensively indicate that the motor feedback involves intricate brain activity. Results from functional magnetic resonance imagining mostly revealed that the motor cortices (e.g., precentral gyrus and postcentral gyrus) [10, 11], basal ganglia [12], and visual cortices [13, 14] exhibit functional prominence for varied experimental conditions of motor feedback, such as precision versus power force grip [10, 13], force magnitude [15], duration of maintained force [16], feedback frequency [17], and maturation of force control [18]. The involvement of these brain areas mainly came from the investigations on a block paradigm which is intermitted periodically (such as 30 s); however, the motor feedback in practice, for example, when driving a car, usually lasts for several minutes/hours. During such long-lasting feedback, sustained attention also plays important roles in motor control [19, 20]. Thus, Dong and his colleagues proposed a continuous performing paradigm for the fMRI investigation of the motor feedback and revealed the altered brain activity in the visual cortex and the areas of the default mode network, for example, posterior cingulate cortex, while comparing real and sham feedback conditions [21].

Recently, frequency-dependent characteristics of brain activity have been reported by more and more fMRI investigations [22–25]. Some separate frequency bands of brain activity such as Slow-6 (0.0–0.01 Hz) [22], Slow-5 (0.01–0.027 Hz), Slow-4 (0.027–0.073 Hz), Slow-3 (0.073–0.198 Hz) [23, 24], and Slow-2 (0.198–0.25 Hz) [23] are generated with specific properties and physiological functions. The frequency-dependent analysis of brain activity exhibits clinical usefulness for quantification and detection of the functional pathological changes in brain disorders such as Parkinson's disease [25]. Although these brain disorders could be treated clinically with the motor feedback, the brain activity of motor feedback remains to be understood in different subfrequency bands.

Brain activity measured with the fMRI signal exhibits the local synchronization of the time courses of neighboring voxels, which could be assessed through the measurement of regional homogeneity (ReHo). Therefore, the present study aims to examine the local synchronization in the subfrequency bands of Slow-6, Slow-5, Slow-4, Slow-3, and Slow-2 during motor feedback. Conventional block paradigm involves the periodical intermission that does not allow frequency-dependent analysis. Thus, we performed the fMRI experiment by employing a continuous paradigm, that is, continuous feedback of finger force. Then, the differences in ReHo between real and sham feedback conditions were investigated in the subfrequency bands.

## 2. Materials and Methods

### 2.1. Participants

Forty-three right-handed college students participated in the study (22.7 ± 1.6 years, range 19–25; 23 females). No participant had the histories of brain injury, neurological illness, or psychiatric disorders. Five subjects were excluded due to the malfunction of experimental equipment or excessive head motion (head motion was >2 mm translation or >2° rotation in any direction), and, at last, data from 38 subjects (mean age, 22.3 ± 1.6 years; 19 females) were involved in the further analysis. All experiments conducted in this study were approved by the Institutional Review Board of the National Key Laboratory of Cognitive Neuroscience, Beijing Normal University. All of the subjects gave written informed before scanning.

### 2.2. Experimental Design

The experimental procedure has been reported in our recent study [21]. Each participant first underwent a scanning of resting state for adapting to the fMRI environment. Then, two scanning sessions, one for continuous real feedback and one for continuous sham feedback, were performed. Each session lasted for 8 min, and the order of the two sessions was counterbalanced across all participants. In the session of real feedback, the participants gripped a pressure sensor between the right index finger and thumb. This sensor is one module of an MRI-compatible physiological multichannel analyzer (model MP150, BIOPAC Systems, Inc., Goleta, CA). The sampling frequency was 250 Hz and the pressure sensitivity was 0.01 cm H_2_O. The pressure was recorded by a sensor via an airtight tube, and the force of pressure was synchronously fed back to the participant via a projector. At the same time, each participant was requested to continuously maintain the pinch force at 20 cm H_2_O as far as possible according to the visual feedback. This target force was set in order to reduce the possibility of muscular fatigue for each subject [26]. In the session of sham feedback, participants were also asked to maintain the pinch force at 20 cm H_2_O as far as possible, and the visual feedback they received came from the performance of another participant in the session of real feedback. The aim of this procedure was to minimize the difference in visual presentation between real and sham feedback sessions. Because sham feedback of pinch force could be easily detected by the subject, we informed participants of this fact in advance and requested them to watch the feedback while keeping their own performance unaffected. Before each session, the participants had a short training period.

### 2.3. Image Acquisition

Brain scans were performed at the MRI Center of Beijing Normal University using a 3.0-T Siemens whole-body MRI scanner. A single-shot T2^∗^-weighted, gradient-echo EPI sequence was used for functional imaging acquisition with the following parameters: TR/TE/flip angle = 2000 ms/30 ms/90°, acquisition matrix = 64 × 64, field of view (FOV) = 200 × 200 mm^2^, and thickness/gap = 3.5/0.7 mm. Thirty-three axial slices parallel to the AC-PC line were obtained in an interleaved order to cover the entire cerebrum and cerebellum. Then a T1-weighted sagittal three-dimensional magnetization-prepared rapid gradient-echo (MPRAGE) sequence was acquired (128 sagittal slices, thickness/gap = 1.33/0 mm, in-plane resolution = 256 × 192, TR = 2530 ms, TE = 3.39 ms, inversion time = 1100 ms, flip angle = 7°, and FOV = 256 × 256 mm^2^).

### 2.4. Data Analysis

#### 2.4.1. Preprocessing

The preprocessing was carried out using the Data Processing Assistant for Resting-State fMRI (DPARSF) [27] which is based on the Statistical Parametric Mapping (SPM8) (http://www.fil.ion.ucl.ac.uk/spm/) and Resting-State fMRI Data Analysis Toolkit (REST) [28] (http://www.restfmri.net/). For each subject, the first 10 time points of the functional data of real/sham feedback were discarded to allow for signal stabilization. These images were further corrected for intravolume acquisition time delay between slices and intervolume geometrical displacement due to head movement. Then, all images were normalized to the standard Montreal Neurological Institute (MNI) template (resampled into 3 × 3 × 3 mm^3^) via parameters of individual structural image spatial normalization based on unified segmentation [29]. Six head motion parameters (three rigid body translations and three rotations) were regressed out from the fMRI data, and the linear trends were removed from the time courses of the voxels in each image. According to previous investigations [22–25], we used band-pass filtering to subdivide the whole detectable frequency range (0–0.25 Hz) into five subfrequency bands, namely, Slow-6 (0.0–0.01 Hz), Slow-5 (0.01–0.027 Hz), Slow-4 (0.027–0.073 Hz), Slow-3 (0.073–0.198 Hz), and Slow-2 (0.198–0.25 Hz). Then, for each subfrequency band, the filtered functional data were further assessed through a voxelwise measurement of the regional homogeneity (ReHo).

#### 2.4.2. Regional Homogeneity (ReHo) Analysis

ReHo is an analytic method widely used in resting-state fMRI studies. It is a voxelwise measure of the brain activity by examining the synchronization of the time courses of a certain voxel and its adjacent neighboring voxels [30].

The ReHo analysis employs Kendall's coefficient of concordance (KCC) to measure the local synchronization of the time courses of neighboring voxels as follows [30]:

(1) $$\begin{array}{c} W=\frac{\sum {\left(R_{i}\right)}^{2}-n{\left(\overset{-}{R}\right)}^{2}}{\left(1/12\right)K^{2}\left(n^{3}-n\right)}, \end{array}$$

where *W* is the KCC among given voxels, ranged from 0 to 1; *R*_*i*_ is the sum rank of the *i*th time point; $\overset{-}{R}=((n+1)K/2)$ is the mean of the *R*_*i*_'s; *K* is the number of time courses within a measured cluster (here, *K* = 27, one given voxel plus the number of its neighbors); *n* is the number of ranks. The KCC was calculated for each 27 nearest neighboring voxels in a voxelwise manner and the KCC value was assigned to the central voxel of each 27-voxel cluster. For each subfrequency band, the ReHo analysis was conducted using DPARSFA. Individual ReHo image during real/sham feedback was generated within a whole-brain mask and nonbrain areas are excluded. The whole-brain mask was provided in REST [28]. The individual ReHo image for each frequency band during real/sham feedback was then smoothed with a 6 × 6 × 6 mm full-width-at-half-maximum (FWHM) Gaussian kernel. Then, a two-way repeated measures analysis of variance (ANOVA) was performed with factors of the feedback condition (2 levels, real and sham) and the frequency band (5 levels, i.e., Slow-6, Slow-5, Slow-4, Slow-3, and Slow-2). Then, the resultant F-maps were corrected for multiple comparisons with the threshold of *P* < 0.005 and cluster size >98 voxels, corresponding to a corrected *P* value of <0.05 as determined by AlphaSim (http://afni.nimh.nih.gov/pub/dist/doc/manual/AlphaSim.pdf). For clusters showing significance in the main effect of the feedback condition factor, region of interest (ROI) was defined with a sphere of 6 mm radius which was centered at the peak coordinate. Then, for each ROI, the mean ReHo across all subfrequency bands and subjects was calculated for real and sham feedback conditions, respectively. For clusters showing significant interaction effect between factors of the feedback condition and the frequency band, we also defined ROIs with a sphere of 6 mm radius which was centered at the peak coordinate. ReHo of each ROI was extracted based on the frequency band and the feedback condition of every subject. Then, for each ROI, paired *t*-tests were further performed to examine the difference of ReHo between real and sham feedback in each subfrequency band. The tested results were corrected for multiple comparisons to a significant level of *P* < 0.05 (Bonferroni correction across the five frequency bands).

## 3. Result

**Figure 1:**
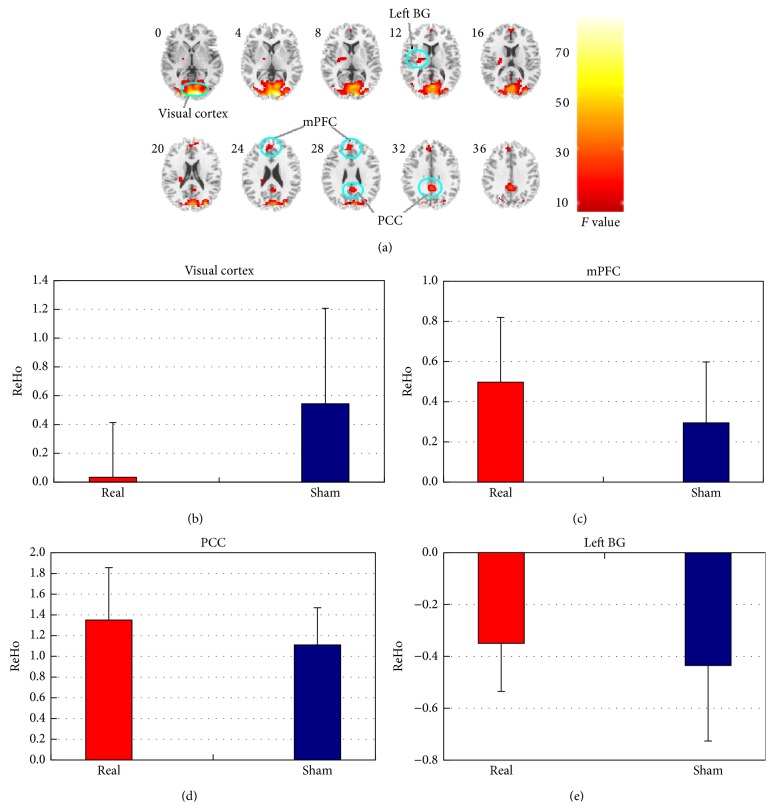
Clusters showing significant main effect of the feedback condition and ReHo of each cluster in all subfrequency bands for real/sham feedback. (a) Slice views of the spatial maps for the main effect of the feedback condition. (b) ReHo of the visual cortex in all subfrequency bands for real/sham feedback; (c) ReHo of the mPFC in all subfrequency bands for real/sham feedback; (d) ReHo of the PCC in all subfrequency bands for real/sham feedback; (e) ReHo of the left BG in all subfrequency bands for real/sham feedback. Red represents real feedback and blue represents sham feedback.

According to the main effect of the feedback condition factor, differences of ReHo between real and sham feedback were distributed in four clusters, including bilateral visual cortex (containing bilateral inferior occipital gyrus, bilateral middle occipital gyrus, and bilateral calcarine), bilateral posterior cingulate cortex (PCC), bilateral medial prefrontal cortex (mPFC), and left BG (mainly located in putamen) (Table 1 and Figure 1(a)). For these clusters, the mean ReHo across all investigated subfrequency bands and subjects were shown in Figures 1(b)–1(e). Visual cortex showed lower ReHo while comparing real feedback with sham feedback (Figure 1(b)). As Figure 1(c) shows, ReHo for the mPFC was greater in real feedback than it was in sham feedback. As to the PCC, real feedback recruited greater ReHo than sham feedback (Figure 1(d)), and, for the left BG, greater ReHo was observed in real feedback as compared with that in sham feedback (Figure 1(e)). The main effect of the frequency band factor was similar to the findings of the previous study [23], and it was not presented here because it is not the focus of the current study.

**Figure 2:**
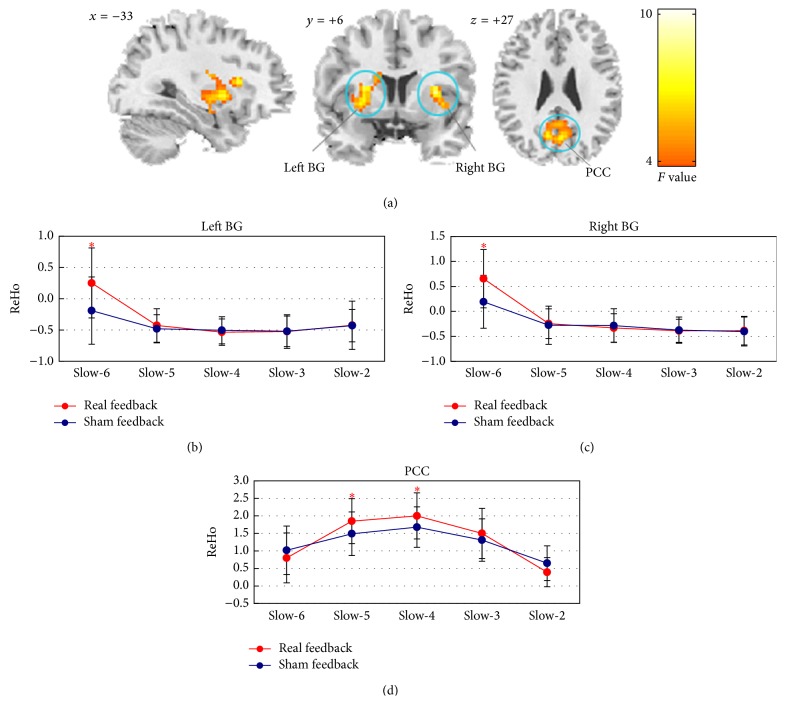
Clusters showing significant interaction effect between factors of the feedback condition and the frequency band and the relevant comparison results of ReHo in different frequency bands between real and sham feedback conditions. (a) Coronal, sagittal, and axial views of the spatial maps for the interaction effect between the feedback condition and the frequency band; (b) changes in ReHo of the left BG across the frequency bands during real and sham feedback; (c) changes in ReHo of the right BG across the frequency bands during real and sham feedback; (d) changes in ReHo of the PCC across the frequency bands during real and sham feedback. Red represents real feedback and blue represents sham feedback. ∗ indicates the significant difference of ReHo between real and sham feedback. The statistical threshold was set at *P* < 0.05, corrected for multiple comparisons.

The interaction effect between factors of the feedback condition and the frequency band was observed in three clusters, that is, the bilateral PCC and both of the left and right basal ganglia (BG) (mainly containing putamen and caudate) (Figure 2(a) and Table 2). For the PCC, real feedback exhibited greater ReHo in the Slow-5 (*t*(37) = 3.71, *P* < 0.005) and Slow-4 (*t*(37) = 3.75, *P* < 0.005) than sham feedback (Figure 2(d)). As Figures 2(b) and 2(c) show, real feedback recruited greater ReHo in the left and right BG than sham feedback and these significant differences were only manifested in Slow-6 (*t*(37) = 4.38, *P* < 0.005 for the left BG and *t*(37) = 4.29, *P* < 0.005 for the right BG).

## 4. Discussion

The present fMRI study investigated the neural substrate of motor feedback using a frequency-dependent analysis. The local synchronization of brain activity was assessed through a voxelwise measurement of ReHo in five separate subfrequency bands ranged from Slow-6 (0.0–0.01 Hz) to Slow-2 (0.198–0.25 Hz). Two intriguing results were observed: (1) as compared with sham feedback, real feedback recruited greater ReHo of the PCC, in Slow-5 and Slow-4; (2) ReHo differences in the left and right BG were mainly manifested in the ultraslow frequency band of Slow-6 which is less concerned in previous neuroimaging explorations.

Few previous investigations have performed fMRI investigations on the neural substrate of motor feedback in different subfrequency bands. This is probably because these investigations mostly employ the periodically blocked paradigm that is not suitable for the frequency-dependent analysis. The present study showed the benefits of the continuous paradigm and frequency-dependent ReHo analysis for examining the frequency-dependent fMRI signal characteristics in the process of task performing. The ReHo differences for the visual cortex and the mPFC were manifested in all but not some specific subfrequency bands. Real feedback recruited lower ReHo in visual cortex than sham feedback. The involvement of the visual cortex is mostly manifested in visually guided motor feedback [13, 14]. It is thought that visual cortex mainly responds to update the visual information and further process the information for the adjustment of the force [13, 21, 31]. In our experiment, participants were requested to maintain their finger force according to real feedback, and, then, the visual processing may be more involved in this condition. Greater ReHo for the mPFC was observed while comparing real feedback with sham feedback. The mPFC is known to be highly sensitive to the sustained attention [32]. Thus, the greater ReHo in real feedback may be linked with the attentional processing of the visual stimuli [33].

The changes of ReHo in the PCC mainly came from Slow-5 and Slow-4 when comparing real feedback with sham feedback. The Slow-5 and Slow-4 cover the frequency range of 0.01–0.073 Hz which is roughly equivalent to the typical low frequency band (0.01–0.08 Hz) [34, 35]. Mostly recruited in the default mode network in the typical low frequency band, the PCC has been identified as the hubs of this network chiefly responsible for attentional lapses and mind wandering [36, 37]. In task state, these areas may play a role as a source of internal interference or noise and were suppressed as deactivation [37, 38], and the deactivation may further induce the elevation of ReHo. Thus, the greater ReHo in the PCC suggested that real feedback requires more suppression of internal interference than sham feedback, and, more importantly, our results indicated that the suppression was potentially associated with the local synchronization of the PCC in Slow-5 and Slow-4.

We observed that ReHo of the left and right BG in Slow-6 is greater for real feedback than it is for sham feedback. Slow-6, as an ultraslow frequency band, is less concerned in previous neuroimaging explorations. In resting state, it was thought that this ultraslow frequency band may reflect very low frequency drift [39]. However, a recent study provides new insights into this issue by showing that oscillations lower than 0.02 Hz contribute more to ReHo in putamen during resting state [40]. Our results support this finding and further indicate that, during motor feedback, the oscillation in the ultraslow frequency band of Slow-6 is critical for ReHo in BG (including bilateral putamen and caudate). The BG is an important brain area for motor feedback. It is suggested that the BG is involved in the planning and parameterizing of motor control [41]. Thus, the ultraslow frequency band of brain oscillation during motor feedback may be associated with these functional roles of BG. Remarkably, the BG disorders such as Parkinson's disease mostly result in the decreased ReHo in the BG [42], and the motor feedback has been employed in the treatment of these disorders exhibiting therapeutic effectiveness [43]. Thus, the ultraslow frequency band of local synchronization during motor feedback may possess the therapeutic value in these clinical practices.

Nevertheless, the current study has some limitations. The sampling rate in the present study (2 s) prevents us from performing the analysis in higher frequency band and we believed that fast sampling should provide more novel findings for motor feedback fMRI studies. Moreover, the results of the present study are restricted to the visual feedback, and the feedback presented in the auditory and sensory forms is commonly employed in practice. Further experimentation and investigation are still required to fully clarify these issues.

## 5. Conclusion

The present fMRI study shed light on the neural substrate of motor feedback by studying the local synchronization in the subfrequency bands ranged from Slow-6 to Slow-2. Using the measurement of ReHo, we found that the five subfrequency bands exhibit distinct contributions to the changes of ReHo between real and sham feedback, which provided novel insights into the neural substrate of motor feedback. The result that changes in left and right BG mainly depended on the ultraslow frequency band of Slow-6, which potentially helps to understand properties of the ultraslow frequency band of local synchronization.

## Figures and Tables

**Table 1:** Clusters showing significant main effect for the feedback condition. The statistical threshold was set at *P* < 0.005, cluster size >98.

Brain regions	L/R	BA	Peak MNI coordinates			
			*x*	*y*	*z*	*F*(1,370)
Inferior/middle occipital Gyrus/calcarine	L/R	17/18	12	−90	0	78.86
PCC	L/R	31	0	−45	33	28.31
mPFC	L/R	9	−6	48	27	18.36
BG	L		−24	−12	12	13.39

**Table 2:** Clusters showing significant interaction effect between the feedback condition and the frequency band. The statistical threshold was set at *P* < 0.005, cluster size >98.

Brain regions	L/R	BA	Peak MNI coordinates			
			*x*	*y*	*z*	*F*(4,370)
PCC	L/R	7/31	0	−66	33	10.08
BG	L		−30	3	0	7.32
BG	R		33	3	6	7.79
